# Roles of a Cysteine Desulfhydrase LCD1 in Regulating Leaf Senescence in Tomato

**DOI:** 10.3390/ijms222313078

**Published:** 2021-12-03

**Authors:** Kangdi Hu, Xiangjun Peng, Gaifang Yao, Zhilin Zhou, Feng Yang, Wanjie Li, Yuqi Zhao, Yanhong Li, Zhuo Han, Xiaoyan Chen, Hua Zhang

**Affiliations:** 1 School of Food and Biological Engineering, Hefei University of Technology, Hefei 230009, China; 2019111433@mail.hfut.edu.cn (X.P.); yaogaifang@hfut.edu.cn (G.Y.); 2019111429@mail.hfut.edu.cn (Y.Z.); yhl719@126.com (Y.L.); kanahan80@163.com (Z.H.); swspcxy@163.com (X.C.); 2 Xuzhou Institute of Agricultural Sciences of the Xuhuai District of Jiangsu Province, Xuzhou 221131, China; zhouzhilinting@163.com (Z.Z.); XZ-YANGFENG@163.com (F.Y.); 3 Key Laboratory of Cell Proliferation and Regulation Biology, Ministry of Education, College of Life Science, Beijing Normal University, Beijing 100875, China; lwj@bnu.edu.cn

**Keywords:** tomato, hydrogen sulfide, cysteine desulfhydrase, leaf senescence, reactive oxygen species

## Abstract

Hydrogen sulfide (H_2_S), a novel gasotransmitter in both mammals and plants, plays important roles in plant development and stress responses. Leaf senescence represents the final stage of leaf development. The role of H_2_S-producing enzyme L-cysteine desulfhydrase in regulating tomato leaf senescence is still unknown. In the present study, the effect of an L-cysteine desulfhydrase LCD1 on leaf senescence in tomato was explored by physiological analysis. *LCD1* mutation caused earlier leaf senescence, whereas *LCD1* overexpression significantly delayed leaf senescence compared with the wild type in 10-week tomato seedlings. Moreover, *LCD1* overexpression was found to delay dark-induced senescence in detached tomato leaves, and the *lcd1* mutant showed accelerated senescence. An increasing trend of H_2_S production was observed in leaves during storage in darkness, while *LCD1* deletion reduced H_2_S production and *LCD1* overexpression produced more H_2_S compared with the wild-type control. Further investigations showed that *LCD1* overexpression delayed dark-triggered chlorophyll degradation and reactive oxygen species (ROS) accumulation in detached tomato leaves, and the increase in the expression of chlorophyll degradation genes *NYC1*, *PAO*, *PPH*, *SGR1*, and *senescence-associated genes* (*SAGs*) during senescence was attenuated by *LCD1* overexpression, whereas *lcd1* mutants showed enhanced senescence-related parameters. Moreover, a correlation analysis indicated that chlorophyll content was negatively correlated with H_2_O_2_ and malondialdehyde (MDA) content, and also negatively correlated with the expression of chlorophyll degradation-related genes and *SAGs*. Therefore, these findings increase our understanding of the physiological functions of the H_2_S-generating enzyme LCD1 in regulating leaf senescence in tomato.

## 1. Introduction

Leaf senescence represents the final stage of leaf development, which is a genetically controlled process [1]. As leaves age, the decomposition of chloroplast is initiated, accompanied by the catabolism of macromolecules including nucleic acids, proteins, and lipids. The decomposed nutrients then transfer to other developing organs, such as young leaves and growing fruit [2]. Chloroplasts constitute approximately 70% of the total proteins in green leaves and chlorophyll degradation causes the first visible signs of leaf senescence [3]. Thus, the coordinated degradation of chlorophyll is crucial for the breakdown of chloroplasts. Terrestrial plants utilize two types of chlorophyll species (i.e., chlorophyll a and chlorophyll b) for photosynthesis [4]. Chlorophyll b has to be converted to chlorophyll a before it can be processed into the degradation pathway and NON-YELLOW COLORING 1 (NYC1) catalyzes the reduction of chlorophyll b to 7-hydroxymethyl chlorophyll a [5]. Chlorophyll a is further decomposed to pheophytin a by Mg-dechelatase NON-YELLOWINGs/STAY-GREENs (NYEs/SGRs) [6]. Pheophytin a is then hydrolyzed by the pheophytinase PPH to generate pheophorbide a which is further catalyzed by oxygenase PAO to produce a red chlorophyll catabolite (RCC) [7]. Moreover, hundreds of *senescence-associated genes* (*SAGs*), whose transcripts increase as leaves age [8,9], are also involved in the regulation of leaf senescence.

Plant hormones are major players influencing each stage of leaf senescence. For instance, ethylene, abscisic acid (ABA), and so on promote leaf senescence, while cytokinins (CKs), gibberellic acid (GA), and so on delay leaf senescence [10,11,12]. Furthermore, leaf senescence-linked events are often associated with the pronounced accumulation of reactive oxygen species (ROS) [13]. Among them, H_2_O_2_ is a well-defined inducer of leaf senescence. Recently, it was reported that transcription factor NAC075 delays leaf senescence by deterring ROS accumulation through directly binding the promoter of the antioxidant enzyme gene *catalase 2* (*CAT*) in *Arabidopsis* [14]. Hydrogen sulfide (H_2_S) is an important gasotransmitter in both animals and plants [15]. H_2_S has not only been implicated in seed germination and root development, but can also enhance plant tolerance to various stresses such as heavy metals, drought, salinity, and cold by enhancing the antioxidant system [16,17,18]. In addition, H_2_S can extend the shelf life of bananas, grapes, strawberries, tomatoes, and so on [19,20,21,22]. The underlining mechanism of H_2_S in alleviating postharvest senescence may involve the activation of the antioxidant system, the inhibition of ethylene synthesis and the signaling pathway, etc.

H_2_S is endogenously produced in a precise and regulated manner. Cysteine degradation by cysteine desulfhydrases (CDes) to the formation of sulfide, ammonia, and pyruvate was believed to be an important source of H_2_S [23]. Plant cells contain different CDes localized in the cytoplasm, plastids, and mitochondria [23]. DES1, an O-acetylserine(thiol)lyase homolog with L-cysteine desulfhydrase activity, regulates cysteine homeostasis in *Arabidopsis* [24]. Recently it was reported that the *des1* mutant was more sensitive to drought stress and displayed accelerated leaf senescence, while the leaves of *OE-DES1* contained adequate chlorophyll levels accompanied by significantly increased drought resistance, suggesting the role of DES1 in regulating leaf senescence [25]. In our previous work, a L-cysteine desulfhydrase named LCD1 was found to localize in the nucleus by a potential nuclear localization signal, and an *lcd1* mutation caused accelerated fruit ripening compared with the wild type [26]. However, whether and how LCD1 participates in leaf senescence or dark-induced senescence are still unknown. In the present study, we focused on the role of endogenous H_2_S-producing enzyme LCD1 during leaf senescence. Moreover, a correlation analysis was applied to investigate the potential relations between H_2_S, ROS, and chlorophyll breakdown.

## 2. Results

### 2.1. Role of LCD1 in Regulating Tomato Leaf Senescence

**Figure 1:**
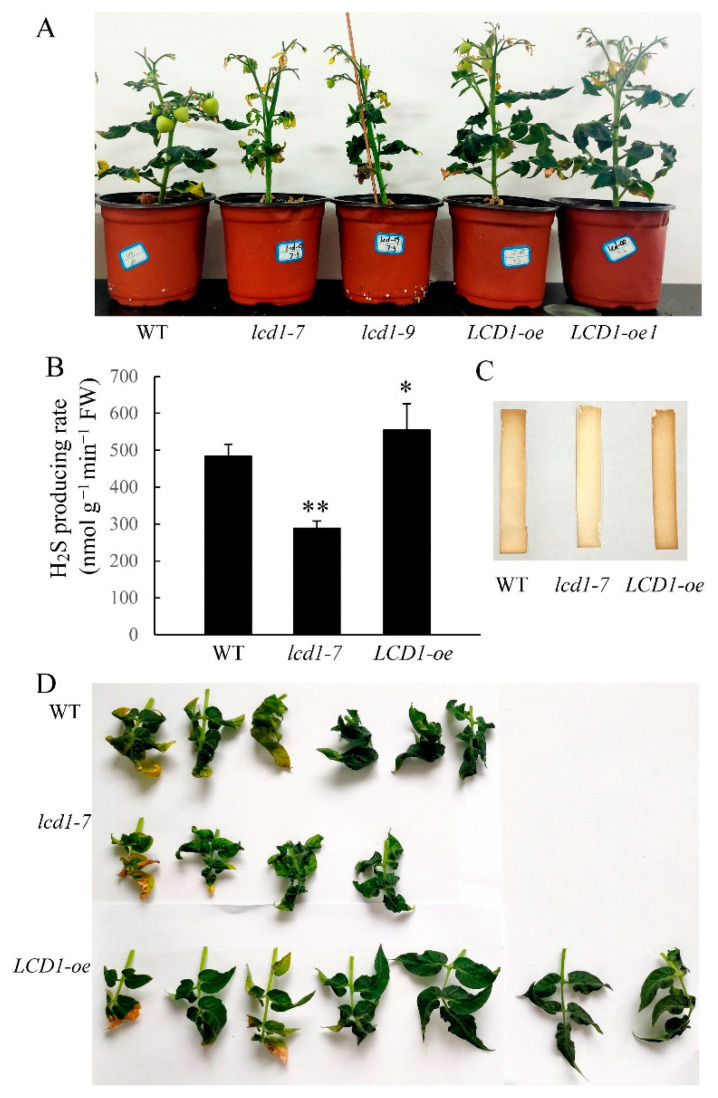
Phenotypic characterization of *lcd1* mutants and *LCD-oe* (over-expression) tomatoes. (**A**) Phenotype of 10-week-old wild-type (WT), *lcd1-7*, *lcd1-9*, *LCD1-oe*, and *LCD1-oe1* plants. (**B**) H_2_S producing rate in the mature leaves from wild type, *lcd1*, and *LCD1-oe* lines of 10 weeks growth. (**C**) H_2_S production from the mature leaves of 10-week-old wild type, *lcd1*, and *LCD1-oe* lines detected by lead acetate H_2_S detection strips (Sigma-Aldrich). (**D**) The leaves of different tomato lines in (**A**) were detached and photographed. Data are means of three biological replicates ± standard deviation (SD). The symbols * and ** stand for *p* < 0.05 and *p* < 0.01 as determined by the Student’s *t*-test, respectively.

To elucidate the possible involvement of LCD1 in regulating leaf senescence, two previously reported tomato lines, *lcd1-7* and *lcd1-9*, with mutations near the PAM sequence were used as *lcd1* mutants, and two lines (hereafter called *LCD1-oe* and *LCD1-oe1*) with an increased expression of *LCD1* under the control of the CaMV *35S* promoter were also used. The overexpression efficacy of five *LCD1* overexpression lines was verified by RT-qPCR as shown in Figure S1 and the lines *LCD1-oe* and *LCD1-oe1*, which showed a higher *LCD1* expression, were used in the present study. To confirm the role of LCD1 in catalyzing H_2_S production, the H_2_S producing rates were determined in leaves of *lcd1* and *LCD1-oe* lines. The data in Figure 1B suggest that *lcd1* leaves had a lower H_2_S producing rate compared with the wild type, while *LCD1* overexpression induced a significantly higher level of the H_2_S producing rate. Besides, H_2_S production was also evaluated by lead acetate H_2_S detection strips, and the results showed that *lcd1* leaves produced less H_2_S and *LCD1* overexpression produced more H_2_S (Figure 1C). After 10 weeks of growth, the *LCD1* mutation caused earlier leaf senescence compared with the wild type. In contrast, *LCD1* overexpression significantly delayed leaf senescence (Figure 1D).

### 2.2. LCD1 Participates in Dark-Induced Senescence

**Figure 2:**
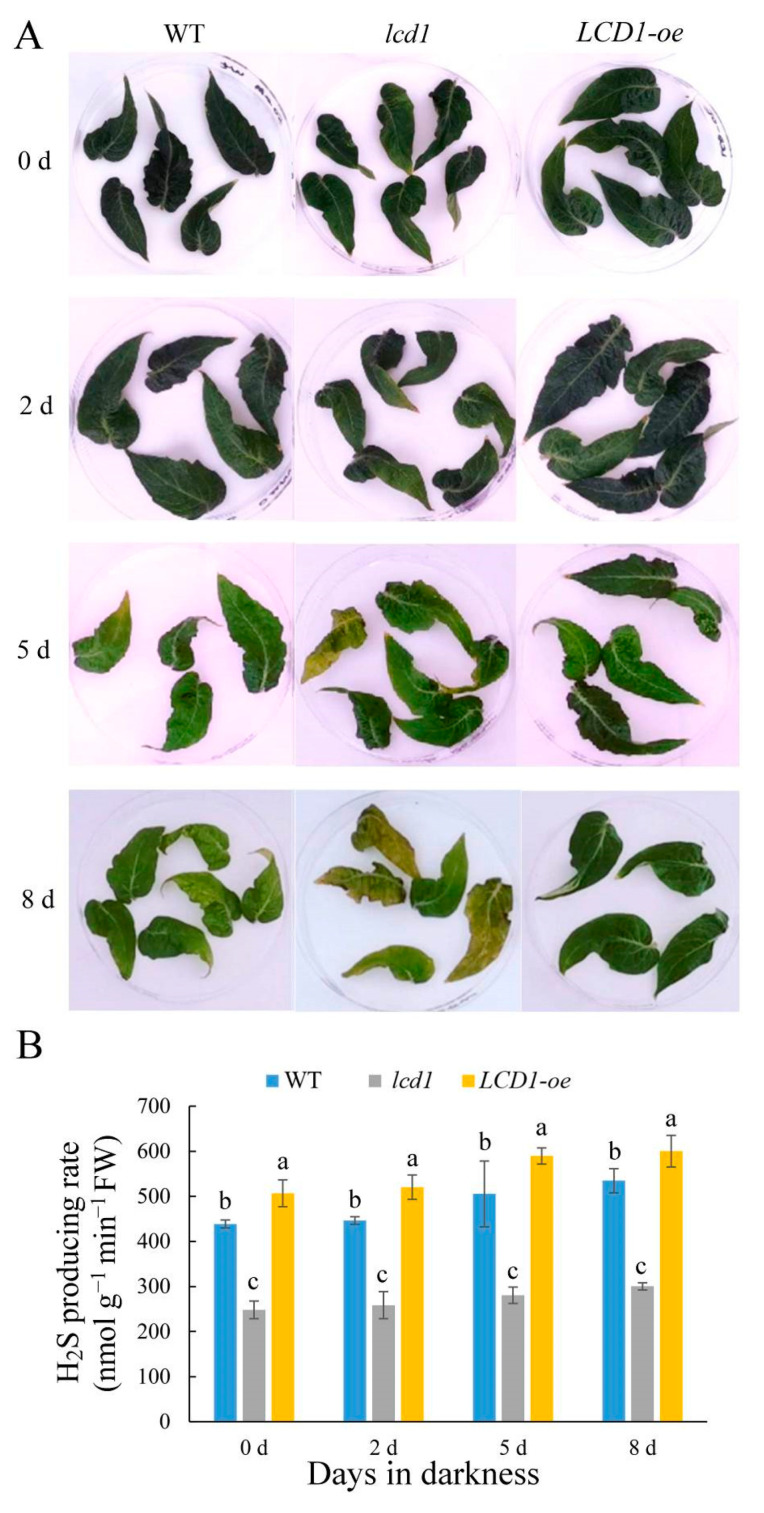
(**A**) Dark-induced senescence symptoms in detached leaves of 6-week-old wild-type (WT), *lcd1*, and *LCD1-oe* tomatoes for up to 8 days. (**B**) H_2_S producing rate in detached leaves of 6-week-old wild-type (WT), *lcd1*, and *LCD1-oe* tomatoes stored in darkness for up to 8 days. Different letters above the columns stand for significant difference between two values (*p* < 0.05) at the same time point.

Leaf senescence is an important phenomenon in the growth and development of plant leaves, and darkness is widely used as a tool to induce senescence in detached leaves. To study the role of LCD1 in dark-induced senescence, the mature leaves without visible senescence of 6-week-old wild type, *lcd1* mutant, and *LCD1-oe* were stored in darkness for 8 days. As shown in Figure 2A, *lcd1* showed the obvious syndrome of the leaf yellowing phenotype after 5 and 8 days in dark stress, whereas *LCD1* overexpression still maintained the green phenotype. To study the kinetics of tomato leaf H_2_S production during senescence, H_2_S production in the leaves at different developmental stages—young leaves (YL), mature leaves (ML), senescent leaves (SL), and late senescent leaves (LS)—was evaluated and the H_2_S detection strips showed browning with senescence, suggesting H_2_S production increased during leaf senescence (Figure S2). Moreover, H_2_S production in leaves of wild-type (WT), *lcd1*, and *LCD1-oe* tomatoes were also determined during dark-induced senescence (Figure 2B). Generally, an increasing trend of H_2_S production was observed in all samples during storage, while *LCD1-oe* leaves showed a higher H_2_S production compared with the wild-type control. In addition, the *lcd1* mutant produced a significantly lower level of H_2_S compared with the wild type. Therefore, it can be concluded that *LCD1* deletion caused a lower H_2_S release and the attenuated H_2_S release may cause an accelerated senescence in the *lcd1* mutant. Overall, the present results indicate that *LCD1* plays a negative role in leaf senescence in both developmental and dark-induced senescence.

### 2.3. Effect of LCD1 on Dark-Triggered Chlorophyll Degradation and Reactive Oxygen Species Accumulation in Detached Tomato Leaves

**Figure 3:**
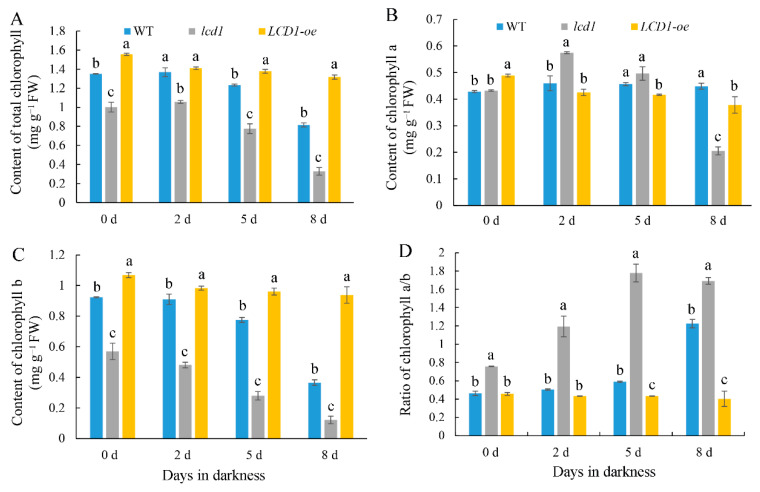
Changes in the contents of (**A**) total chlorophyll, (**B**) chlorophyll a, (**C**) chlorophyll b, and (**D**) the ratio of chlorophyll a/b in dark-stored detached leaves of 6-week-old wild-type (WT), *lcd1*, and *LCD1-oe* tomatoes for 0, 2, 5, and 8 days. Data are means of three biological replicates ± standard deviation (SD). Different letters above the columns stand for significant difference between two values (*p* < 0.05) at the same time point.

Chlorophyll degradation is the one of the most significant changes during leaf senescence; thus, chlorophyll contents were determined in wild-type, *lcd1* mutant, and *LCD1-oe* leaves during dark-induced senescence. As shown in Figure 3A, the content of total chlorophyll in the wild type decreased gradually during storage in darkness for 8 days, whereas the content of chlorophyll in the *lcd1* mutant showed an obvious decrease on days 5 and 8 under darkness, and the value on day 8 was about 32.6% of the initial value on day 0. In contrast, *LCD1* overexpression maintained a relatively higher chlorophyll content compared with the wild type and the *lcd1* mutant on days 5 and 8 under darkness. After 8 days in darkness, the chlorophyll content in *LCD1* overexpression decreased to 84.6% of the initial value, suggesting the role of *LCD1* in delaying dark-induced senescence. As shown in Figure 3B, there were minor changes in the chlorophyll a content between different groups during storage. Moreover, only a slight decrease in chlorophyll a was observed during dark-induced senescence, except for a significant decline found in *lcd1* on day 8. Figure 3C shows the change pattern of chlorophyll b content in wild type, *lcd1* mutant, and *LCD1-oe* during dark-induced senescence. With the increase of storage days, the chlorophyll b content decreased in each group. At day 0, chlorophyll b content in *lcd1* leaves was about 53.4% of that in the *LCD1-oe* group, and decreased to 21.3% on day 8 compared with the value on day 0. Furthermore, the ratio of chlorophyll a/b was also evaluated in dark-stored detached leaves of wild-type, *lcd1*, and *LCD1-oe* tomatoes for 0, 2, 5, and 8 days. As shown in Figure 3D, the ratio of chlorophyll a/b in WT and *lcd1* mutant leaves increased during storage, while *LCD1* deletion caused the highest ratio compared with other groups. In contrast, the ratio of chlorophyll a/b in *LCD1* overexpression almost remained unchanged. The above results indicate that the *lcd1* mutation accelerated dark-induced leaf senescence and *LCD1* overexpression delayed leaf yellowing and chlorophyll degradation.

**Figure 4:**
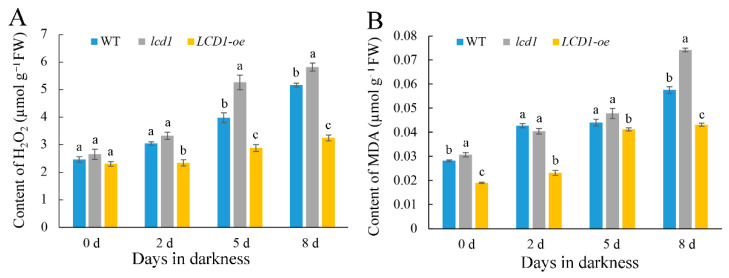
Changes in the contents of (**A**) H_2_O_2_ and (**B**) malondialdehyde (MDA) in dark-stored detached leaves of 6-week-old wild-type (WT), *lcd1*, and *LCD1-oe* tomatoes for 0, 2, 5, and 8 days. Data are means of three biological replicates ± standard deviation (SD). Different letters above the columns stand for significant difference between two values (*p* < 0.05) at the same time point.

Leaf senescence is usually associated with the excessive accumulation of ROS; therefore, the levels of H_2_O_2_ and malondialdehyde (MDA) were monitored in wild-type, *lcd1* mutant, and *LCD1-oe* leaves during dark-induced senescence. As shown in Figure 4A, there was no significant difference in H_2_O_2_ content between the different groups on day 0. During the dark-induced senescence, the H_2_O_2_ content in each group showed an increasing trend, of which the *lcd1* group increased the fastest, followed by the wild-type and *LCD1-oe* group. However, H_2_O_2_ content in the *LCD1-oe* group increased slowly compared with other groups. As shown in Figure 4B, the change of MDA content among the groups also showed a similar trend to H_2_O_2_. The content of MDA in *lcd1* leaves during storage was the highest compared with other groups, and the lowest MDA content was observed in *LCD1-oe* leaves. Therefore, it can be concluded that the overexpression of *LCD1* could reduce the accumulation of ROS and MDA in leaves under dark-triggered senescence.

### 2.4. Effect of LCD1 on the Expressions of Genes Related to Chlorophyll Degradation in Detached Tomato Leaves

**Figure 5:**
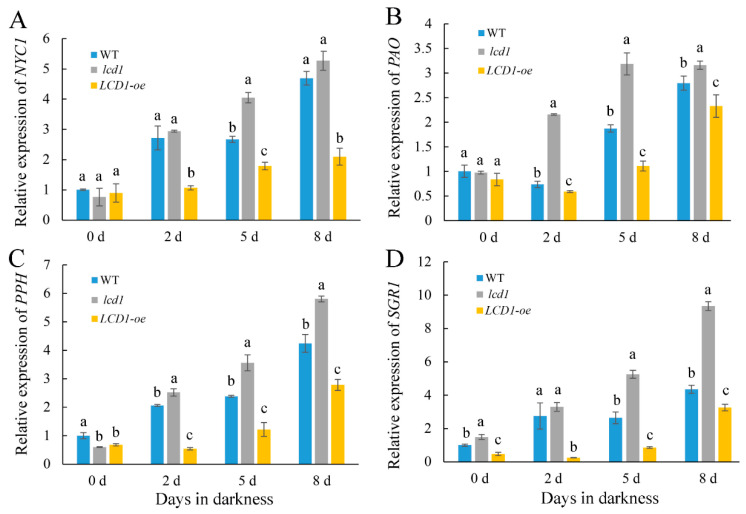
Changes in the gene expressions of chlorophyll degradation related genes: (**A**) *NYC1*, (**B**) *PAO*, (**C**) *PPH*, and (**D**) *SGR1* in detached leaves of 6-week-old wild-type (WT), *lcd1*, and *LCD1-oe* tomatoes stored in darkness for 0, 2, 5, and 8 days. Data are means of three biological replicates ± standard deviation (SD). Different letters above the columns stand for significant difference between two values (*p* < 0.05) at the same time point.

Chlorophyll degradation marks the senescence stage of leaves. In order to explore the molecular mechanism of the differences in chlorophyll content of *lcd1*, *LCD1-oe*, and wild-type leaves during dark-induced senescence, the expression levels of key genes *NYC1*, *PAO*, *PPH*, and *SGR1* in the chlorophyll degradation pathway were analyzed by RT-qPCR. The present data showed *NYC1* was transcriptionally induced during dark-induced senescence in all groups (Figure 5A). In accordance with the early senescence phenotype of the *lcd1* mutant and late senescence in *LCD1-oe* leaves, the expression of *NYC1* was significantly higher in the *lcd1* mutant and was less expressed in *LCD1-oe* leaves during dark storage. Three other genes—*PAO* (Figure 5B), *PPH* (Figure 5C), and *SGR1* (Figure 5D)—were also analyzed at the transcriptional level in wild-type, *lcd1* mutant, and *LCD1-oe* leaves during dark-induced senescence and similar changes to that of the *NYC1* expression were observed. The higher expression of *PAO*, *PPH*, and *SGR1* in *lcd1* and lower expression in *LCD1-oe* again supported the role of *LCD1* in delaying leaf senescence. The results suggest that LCD1 may delay the chlorophyll degradation by down-regulating the transcription of key genes in the chlorophyll degradation pathway.

### 2.5. Effect of LCD1 on the Expressions of SAGs in Detached Tomato Leaves

**Figure 6:**
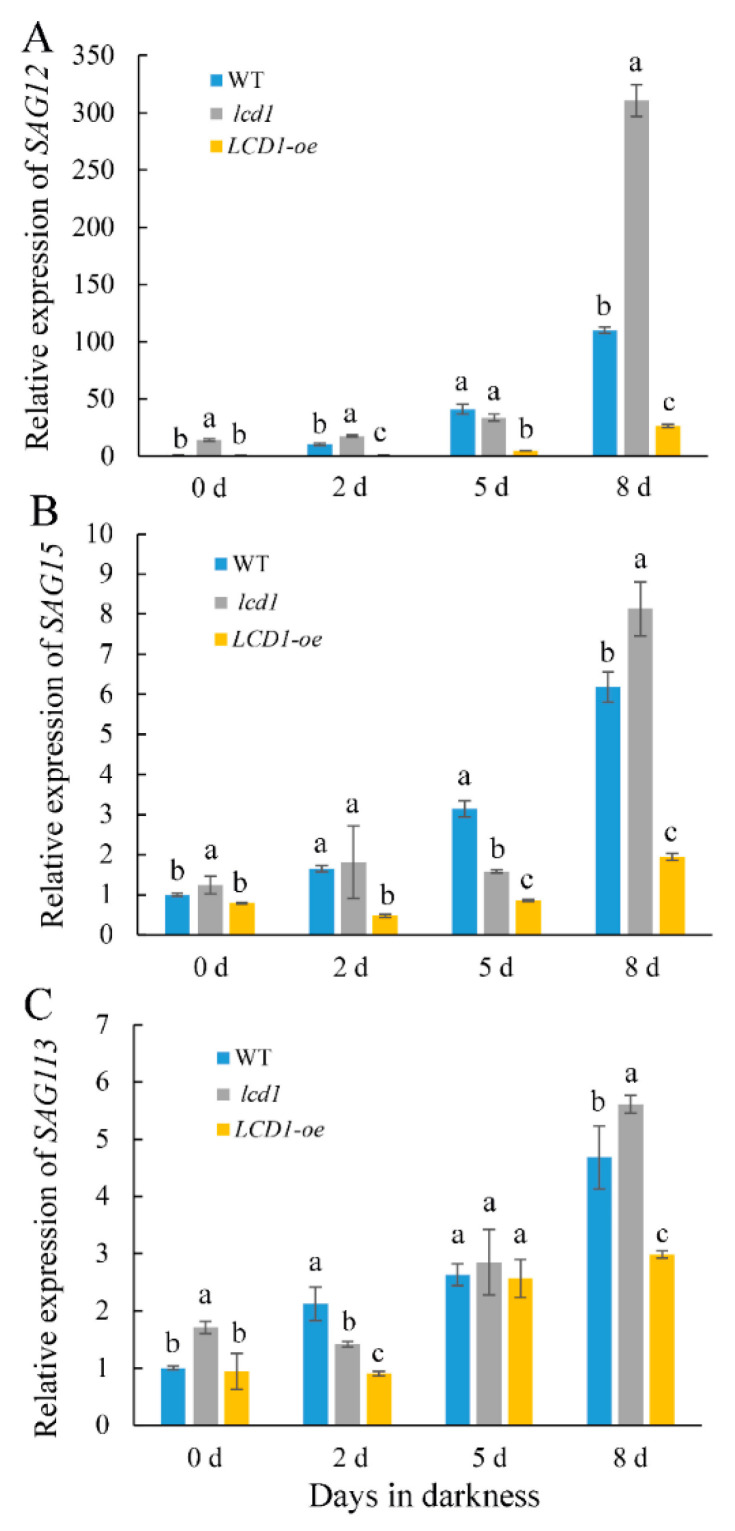
Changes in the gene expressions of senescence-related genes: (**A**) *SAG12*, (**B**) *SAG15*, and (**C**) *SAG113* in detached leaves of 6-week-old wild-type (WT), *lcd1*, and *LCD1-oe* tomatoes stored in darkness for 0, 2, 5, and 8 days. Data are means of three biological replicates ± standard deviation (SD). Different letters above the columns stand for significant difference between two values (*p* < 0.05) at the same time point.

To further analyze the senescence-alleviating role of LCD1, we conducted an RT-qPCR analysis to evaluate the expression patterns of senescence-associated genes (*SAGs*) in *lcd1*, *LCD1-oe*, and wild-type leaves during dark-induced senescence. As shown in Figure 6, *SAG12*, *SAG15*, and *SAG113* were transcriptionally induced during dark-induced senescence. Compared with *SAG15* and *SAG113*, *SAG12* showed more fold changes during leaf senescence, which was 109.6 times in the wild type on day 8 compared with day 0 (Figure 6A). In accordance with the early senescence phenotype of the *lcd1* mutant and late senescence in *LCD1-oe* leaves, the expression of the three *SAGs* was significantly higher in the *lcd1* mutant and was less expressed in *LCD1-oe* leaves during dark storage, especially on day 8.

### 2.6. Correlation Analysis of Different Leaf Physiological Indexes and Senescence-Related Gene Expression

**Figure 7:**
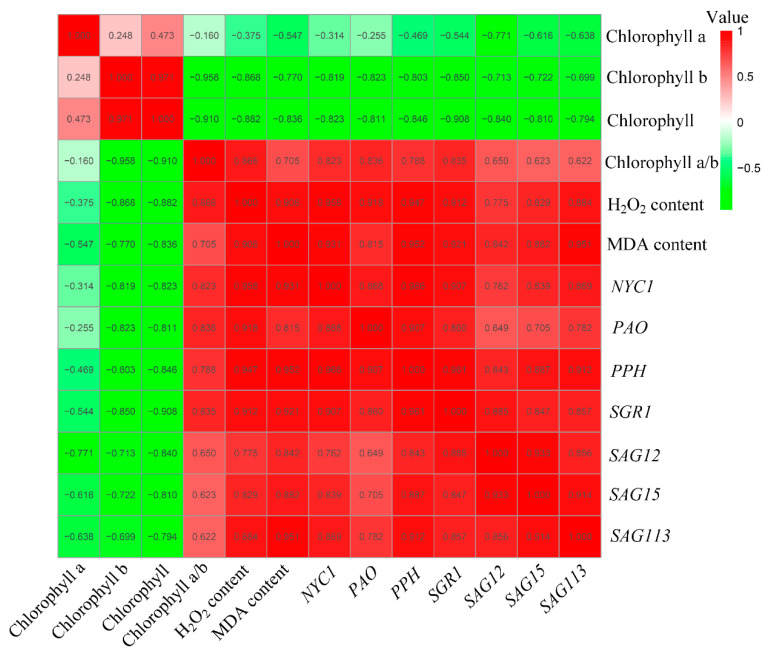
Correlation analysis among the parameters of chlorophyll, chlorophyll a, chlorophyll b, chlorophyll a/b, H_2_O_2_, malondialdehyde (MDA), and gene expressions of *NYC1*, *PAO*, *PPH*, *SGR1*, *SAG12*, *SAG15*, and *SAG113* in detached leaves of 6-week-old wild-type, *lcd1*, and *LCD1-oe* tomatoes stored in darkness for 0, 2, 5, and 8 days.

The correlation among total chlorophyll, chlorophyll a, chlorophyll b, chlorophyll a/b, H_2_O_2_, and MDA content and the gene expression of *NYC1*, *PAO*, *PPH*, *SGR1*, *SAG12*, *SAG15*, and *SAG113* was analyzed to investigate the potential relations among the indexes. As shown in Figure 7, chlorophyll content was negatively correlated with H_2_O_2_, MDA content, and with the expression of chlorophyll degradation-related genes *NYC1*, *PAO*, *PPH*, and *SGR1* and senescence-related genes *SAG12*, *SAG15*, and *SAG113*. Moreover, total chlorophyll and chlorophyll b showed a higher negative correlation to ROS content and senescence-related gene expression in comparison to chlorophyll a. The contents of H_2_O_2_ and MDA were positively correlated with the expression levels of *NYC1*, *PAO*, *PPH*, *SGR1*, *SAG12*, *SAG15*, and *SAG113*. Among them, the expressions of *PPH* and *NYC1* were highly positively correlated (r = 0.966), and the total chlorophyll content was highly negatively correlated with the H_2_O_2_ content (r = −0.882). Moreover, chlorophyll a/b also showed a significant positive correlation with H_2_O_2_ and MDA content. Through these analyses, the positive correlation between H_2_O_2_/MDA content and senescence-related gene expressions indicates that they may act synergistically to accelerate the senescence process of leaves.

### 2.7. Principal Component Analysis of Different Leaf Physiological Indexes and Senescence-Related Gene Expression

**Figure 8:**
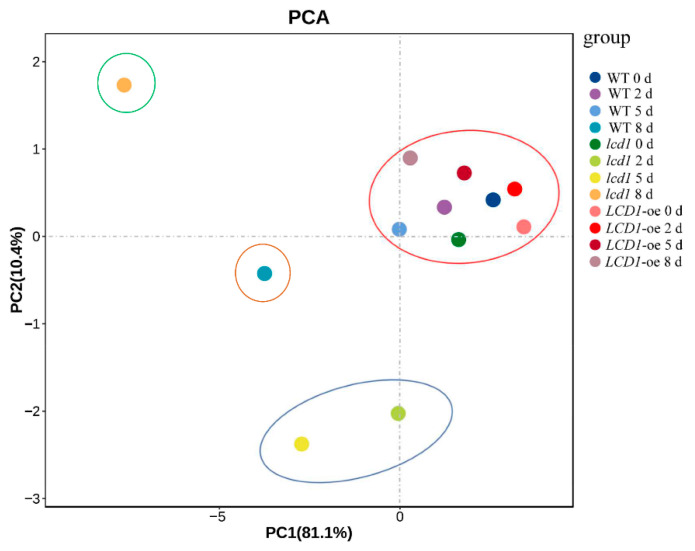
Principal component analysis based on the parameters of chlorophyll, chlorophyll a, chlorophyll b, chlorophyll a/b, H_2_O_2_, malondialdehyde (MDA), and gene expressions of *NYC1*, *PAO*, *PPH*, *SGR1*, *SAG12*, *SAG15*, and *SAG113* in detached leaves of 6-week-old wild-type, *lcd1*, and *LCD1-oe* tomatoes stored in darkness for 0, 2, 5, and 8 days.

The principal component analysis (PCA) was performed based on the data of chlorophyll, H_2_O_2_, and MDA content and the expressions of chlorophyll degradation-related genes and *SAGs*. As shown in Figure 8, PC1 and 2 contributed to 81.1% and 10.4% of the variability of the data, respectively. It can be seen that *lcd1* 2 d and *lcd1* 5 d clustered together, and *lcd1* 8 d was distributed separately from other groups. The variety showing the highest positive load value in the direction of PC1 was *LCD1-oe* 0 d and the variety that showed the lowest load value in the direction of PC2 was *lcd1* 5 d. Therefore, it could be concluded that a decrease in the endogenous H_2_S content in the *lcd1* mutant caused significant changes during dark-induced senescence compared with other groups.

## 3. Discussion

H_2_S, a multifunctional signaling molecule in plants, was found to alleviate the postharvest senescence of broccoli, banana, grape, and tomato [19,20,21,22,27]. Furthermore, H_2_S is implicated in suppressing the chlorophyll degradation of detached leaves of *Arabidopisis* by regulating a dark-dependent reaction [13]. DES1, an O-acetylserine(thiol)lyase homolog with L-cysteine desulfhydrase activity, regulates cysteine homeostasis in *Arabidopsis* [24]. Recently it was reported that a *des1* mutant displayed accelerated leaf senescence, while the leaves of *OE-DES1* contained adequate chlorophyll levels, suggesting the role of DES1 in regulating leaf senescence [25]. In our previous work, mutation of an L-Cys desulfhydrase named LCD1 caused accelerated fruit ripening compared with the wild type [26]. In the present study, to elucidate the possible involvement of LCD1 in regulating leaf senescence, two previously reported tomato lines, *lcd1-7* and *lcd1-9*, with mutations near the PAM sequence were used as *lcd1* mutants, and two lines (hereafter called *LCD1*-oe and *LCD1*-oe1) with an increased expression of *LCD1* under the control of the CaMV 35S promoter were also used. To confirm the role of *LCD1* in catalyzing H_2_S production, the H_2_S producing rates were determined in leaves of the *lcd1* and *LCD1*-oe lines; *lcd1* leaves showed a lower H_2_S producing rate compared with the wild type, while *LCD1* overexpression induced a significantly higher level of the H_2_S producing rate, suggesting the efficiency of LCD1 in producing H_2_S.

Leaf senescence is a highly programmed degeneration process during the final stage of leaf development. To study the role of H_2_S-producing enzyme LCD1 in regulating natural leaf senescence, we compared 10-week old tomatoes of *lcd1* mutants, and the two lines of *LCD1-oe* with the enhanced expression of *LCD1*, and found that *lcd1* developed more senescence syndrome while *LCD1* overexpression maintained more functional leaves.

Prolonged darkness is often used to initiate rapid and synchronous senescence in detached leaves [13]. The roles of LCD1 on dark-induced senescence were evaluated in tomato leaves. The *lcd1* mutant leaves showed an obvious syndrome of the leaf yellowing phenotype after 5 and 8 days in dark stress, whereas *LCD1* overexpression still maintained the green phenotype (Figure 2A). All this evidence suggests the role of H_2_S in alleviating the dark-induced senescence of detached leaves. In accordance with the phenotype of accelerated senescence in the *lcd1* mutant and delayed senescence in *LCD1-oe* leaves, chlorophyll decreased significantly in *lcd1*, but the decrease was attenuated in *LCD1-oe* leaves. Interestingly, we found that chlorophyll b may contribute more to the decrease in total chlorophyll compared with chlorophyll a (Figure 3). By analyzing the ratio of chlorophyll a/b during leaf storage, it was found that the ratio in *LCD1* deletion increased significantly, implying that more chlorophyll b was decomposed to chlorophyll a in the *lcd1* mutant. Moreover, the ratio in *LCD1* overexpression almost remained unchanged, suggesting the significant impact of H_2_S content on the ratio of chlorophyll a/b. Then, the expression levels of key genes *NYC1*, *PAO*, *PPH*, and *SGR1* in the chlorophyll degradation pathway were analyzed by RT-qPCR. It was found that *NYC1*, *PAO*, *PPH*, and *SGR1* transcript abundance increased during darkness in all groups, especially in *lcd1* mutant leaves, whereas this response was significantly inhibited by *LCD1* overexpression (Figure 5). Senescence-associated gene (*SAG*) *12*, *15*, and *113* are widely used as molecular markers for leaf senescence [28] and their transcriptions were also analyzed in detached tomato leaves. In agreement with the phenotype, significant increases in *SAGs* expression were observed in all groups, especially in *lcd1* mutant leaves, and the increase was greatly attenuated by *LCD1* overexpression (Figure 6). The results suggest that LCD1 may delay the chlorophyll degradation by down-regulating the transcription of key genes in the chlorophyll degradation pathway and *SAGs*.

Leaf senescence is often associated with the pronounced accumulation of ROS [13]. Recently, it was reported that transcription factor NAC075 delays leaf senescence by deterring ROS accumulation through directly activating the expression of the antioxidant enzyme gene *catalase 2* (*CAT*) in *Arabidopsis* [14]. To unveil the relations between H_2_S and ROS metabolism in leaf senescence, H_2_O_2_ and MDA contents were determined during leaf senescence in darkness. During the dark-induced senescence, the H_2_O_2_ and MDA content in each group showed an increasing trend, but the overexpression of *LCD1* could reduce the accumulation of ROS and MDA in leaves under dark-triggered senescence. Furthermore, the correlation analysis indicated that the ROS and MDA content showed a higher negative correlation to total chlorophyll and chlorophyll b in comparison to chlorophyll a. The contents of H_2_O_2_ and MDA were positively correlated with the expression levels of *NYC1*, *PAO*, *PPH*, *SGR1*, *SAG12*, *SAG15*, and *SAG113*. The positive correlation between H_2_O_2_ and MDA content and senescence-related genes indicates that they may act synergistically to accelerate the senescence process of leaves, whereas *LCD1* overexpression delayed leaf senescence by inhibiting ROS accumulation and senescence-related gene expressions. Interestingly, increasing H_2_S production was observed in natural senescence leaves (Figure S2). We hypothesize that endogenous H_2_S production was activated to counteract the effect of increasing ROS in senescence leaves. In our previous reports, exogenous H_2_S fumigation delayed the postharvest senescence of broccoli in a dose-dependent manner and H_2_S maintained higher levels of chlorophyll, carotenoids, anthocyanin, and ascorbate, suggesting the role of H_2_S in delaying the postharvest senescence of broccoli [21]. Moreover, H_2_S treatment effectively alleviates ethylene-induced banana peel yellowing and fruit softening [22]. The above results suggest that H_2_S is an effective signal in delaying the postharvest senescence of fruits and vegetables. In the present research, an increasing trend of H_2_S production was observed during leaf senescence, suggesting that H_2_S generation may be activated in response to leaf senescence as ROS metabolites (H_2_O_2_ and MDA) accumulate during dark-induced senescence. Leaf senescence, once initiated, cannot be stopped. Though more H_2_S is produced during leaf senescence, leaves still undergo senescence during storage in darkness. Moreover, compared with the early senescence phenotype of the *lcd1* mutant, *LCD1* overexpression induced more H_2_S production and showed a delayed leaf senescence, clearly suggesting the role of H_2_S in delaying leaf senescence. In all, the data suggest that senescent leaves produced more H_2_S, but reduction in H_2_S production of the *lcd1* mutant caused early senescence in both natural and dark-induced senescence. Moreover, the principal component analysis (PCA) in Figure 8 shows that *lcd1* 2 d and *lcd1* 5 d clustered together, and *lcd1* 8 d was distributed separately from other groups, suggesting that the decreased endogenous H_2_S content in *lcd1* caused significant changes during dark-induced senescence compared with other groups.

## 4. Conclusions

In the present study, the role of a cysteine desulfhydrase LCD1 in regulating leaf senescence in tomato was explored. The *LCD1* mutation caused an earlier leaf senescence, whereas *LCD1* overexpression significantly delayed leaf senescence compared with the wild type in 10-week tomato seedlings. Furthermore, LCD1 was found to play a negative role in dark-induced senescence in detached tomato leaves. Further investigations showed that LCD1 delayed dark-triggered chlorophyll degradation and ROS accumulation in detached tomato leaves, and the increase in chlorophyll degradation and *SAGs* related gene expression was attenuated by *LCD1* overexpression. Moreover, a correlation analysis indicated that chlorophyll content was negatively correlated with H_2_O_2_ and MDA content, and also negatively correlated with the expression of chlorophyll degradation-related genes *NYC1*, *PAO*, *PPH*, and *SGR1* and senescence-related genes *SAG12*, *SAG15*, and *SAG113*. Therefore, these findings increase our understanding of the physiological functions of the H_2_S-generating enzyme LCD1 in regulating leaf senescence in tomato.

## 5. Materials and Methods

### 5.1. Plant Material and Growth Conditions

*Solanum lycopersicum* cv. “Micro-Tom” was used as the control in this study. The mutants *lcd1–7*, which contained a T residue inserted near the PAM sequence, and *lcd1–9*, which had a deletion of G near the PAM as previously reported were used as *lcd1* mutants [26]. The coding sequence of tomato cysteine desulfhydrylase LCD1 (LOC101258894) was obtained from NCBI (http://www.ncbi.nlm.nih.gov/, accessed on 11 September 2018) and the primers including the restriction enzyme sites (LCD1-F: CGCGGATCCTAATCCTAAATGGAACCGGC; LCD1-R: CCGCTCGAGTTCTGAGTGAAGCATCTTAC, the underlines stand for BamHI and XhoI sites, respectively) were used to amplified the coding sequence of *LCD1*. Then, the coding sequence of *LCD1* was inserted into the pBI121 vector at the sites of BamHI and XhoI and transformed into wild-type tomato by *Agrobacterium tumefaciens*, which contained the recombinant *LCD1*-pBI121. The efficiency of *LCD1* overexpression was verified by RT-qPCR. The seeds of tomatoes were grown in a nutrient soil:vermiculite (3:1, *v/v*) in growth pots 10 cm in diameter in an environment-controlled growth room (23 ± 2 °C; 50–70% relative humidity, RH) under 16 h light/8 h dark and 250 mol/m^2^/s light.

### 5.2. Determination of H_2_S Producing Rate and H_2_S Detection in Tomato Leaves

The H_2_S producing rate was measured as described previously [29]. Tomato leaves at 1 g were ground to a fine powder in liquid nitrogen and homogenized in 9 mL of 20 mM Tris-HCl, pH 8.0. After centrifugation at 12,000× *g* for 15 min, the protein content of the supernatant was sampled and the H_2_S producing rate was detected by monitoring the release of H_2_S from L-cysteine in the presence of dithiothreitol (DTT). The assay was performed in a total volume of 1 mL comprising 0.8 mM L-cysteine, 2.5 mM DTT, 100 mM Tris-HCl, pH 8.0, and 100 μL of protein solution. The reaction was incubated for 15 min at 37 °C, and terminated by adding 100 μL of 30 mM FeCl_3_ dissolved in 1.2 N HCl and 100 μL of 20 mM N,N-dimethyl-phenylenediamine dihydrochloride dissolved in 7.2 N HCl. The formation of methylene blue was determined at 670 nm.

The end-point detection of H_2_S production from tomato leaves by lead acetate strips (cat. number WHA2602501A, Sigma, Darmstadt, Germany) were performed as previously described [30]. One gram of fresh tomato leaves was ground to a fine powder in liquid nitrogen and then homogenized in 10 mL of Phosphate Buffered Saline (pH 6.8) supplemented with 10 mM L-cysteine and 10 µM pyridoxal-5′-phosphate (PLP), and then the mixture was placed in petri dishes. The H_2_S detection strips were attached to the inner part of the upper lid of the petri dishes and incubated at 37 °C for 2–5 h until lead sulfide darkening of the strip occurred.

### 5.3. Dark Treatment of Tomato Leaves

For dark-induced leaf senescence experiments, detached mature leaves from 6-week-old wild-type, *lcd1*, and *LCD1-oe* transgenic plants were placed on filter papers which were moistened by 2 mL of sterile water in petri dishes with a 9 cm diameter and the adaxial side facing upwards. The petri dishes which contained 5–6 detached leaves were kept in darkness at 23 °C for 8 days. The leaves were sampled and rapidly frozen in liquid nitrogen and stored at −80 °C until analysis.

### 5.4. Determination of Chlorophyll Content

Tomato leaves at 2 ± 0.01 g were homogenized in liquid nitrogen and subsequently extracted ethanol and 80% (*v/v*) acetone solution in a ratio of 1:1 (*v/v*) according to the method in [31]. The absorbance of the supernatant was read at 663 and 645 nm. The experiments were repeated three biological times, and the chlorophyll levels were expressed as mg/g fresh weight (FW).

### 5.5. Determination of H_2_O_2_ and Malondialdehyde (MDA) Content in Tomato Leaves

The contents of H_2_O_2_ and malondialdehyde (MDA) were assayed as described by Ge et al. [22] and Hu et al. [27]. For the determination of H_2_O_2_ content, 2.0 ± 0.01 g of tomato leaves were homogenized in 3 mL of precooled acetone, and centrifuged at 12,000× *g* for 30 min. The content of H_2_O_2_ was measured by determining the absorbance value at 508 nm. For the determination of MDA content, 2.0 ± 0.01 g of tomato leaf powder was homogenized with 5% trichloroacetic acid, and the supernatant was obtained by centrifugation at 12,000× *g* for 30 min. The absorbance values were measured at 600 nm, 532 nm, and 450 nm, respectively. The experiments were repeated three times, and the contents of H_2_O_2_ and MDA were expressed as µmol/g fresh weight (FW).

### 5.6. Gene Expression Analysis

Total RNA from 0.1 g of tomato leaves was extracted using an Extraction Kit (Tiangen, Beijing, China) and cDNA was synthesized by a reverse transcription kit (PrimeScript RT Master Mix; Takara, Kyoto, Japan). Then the cDNA products were used for gene expression analysis by quantitative polymerase chain reaction (qPCR) performed using a Bio-Rad IQ5 (Hercules, CA, USA). The specific primers used for qPCR were designed based on the coding sequence of the genes as shown in the SGN database (https://solgenomics.net/, accessed on 12 April 2021). *Tubulin* gene expression in control tomato plants was used for the normalization of the data. The experiments were repeated in three technical replicates.

### 5.7. Data Analysis

The statistical analysis of data was based on Student’s *t*-tests. Significant differences were evaluated using multiple pair wise *t*-test comparisons at *p* < 0.05. The correlation among the contents of chlorophyll, H_2_O_2_, MDA, and the expression of chlorophyll degradation related genes and *SAGs* in tomato leaves and the principal component analysis (PCA) of the data above were analyzed by the tools on the OmicShare platform (https://www.omicshare.com/, accessed on 20 November 2021).

## Data Availability

Data are contained within the article or Supplementary Material.

## Supplementary Materials

The following are available online at https://www.mdpi.com/article/10.3390/ijms222313078/s1.
