# Supplementary material for: Roles of a Cysteine Desulfhydrase LCD1 in Regulating Leaf Senescence in Tomato
Source: Int J Mol Sci. 2021 Dec 3;22(23):13078. doi: 10.3390/ijms222313078 (PMC8658025; doi:10.3390/ijms222313078)
Supplement: Supplementary file 1 [file ijms-22-13078-s001.zip › ijms-1495543-supplementary.pdf]

Table S1. Primers used for quantitative RT-PCR.

Primers	Sequence	Gene ID
SGR1 (forward)	GGCTATCTCCCAAACCATCAA	Solyc08g080090
SGR1 (reverse)	ACTCTGCAACAACTTCATCTCT	
PPH (forward)	CAGATGTCGTGGATGGGAAA	Solyc01g088090
PPH (reverse)	GCCTTTAGCTTCACTTGGTAAAC	
PAO (forward)	CCTCATCGTCTTGCTCCTTTAT	Solyc11g066440
PAO (reverse)	GCAGCTTGAGGTATCCTTGT	
NYC1 (forward)	GATCAGGGAAGAGCACTGTATG	Solyc07g024000
NYC1 (reverse)	GAGAACACAGACACCCAAGTAT	
SAG12 (forward)	GCTACACCAACTGAAAACAGG	Solyc02g076910
SAG12 (reverse)	GAAAGGCAGTATCCAAGAGTCC	
SAG15 (forward)	AAGGTTCCAGCCCATCTTG	Solyc03g117950
SAG15 (reverse)	TCACGCAACCCCAATAGTATC	
SAG113 (forward)	AGATGCGAGCTTCAAACTCC	Solyc05g052980
SAG113 (reverse)	AACGGAAAGAGGAATAGCGAC	
LCD1 (forward)	GAGGGACGGTTTAAGAAAGGAG	LOC101258894
LCD1 (reverse)	GAAGAGGAAAGGGCAAATGAAC	
Tubulin (forward)	TAGAGCCTGGTACGATGGATAG	Solyc08g006890
Tubulin (reverse)	CAACTCAGCGCCTTCAGTATAA	

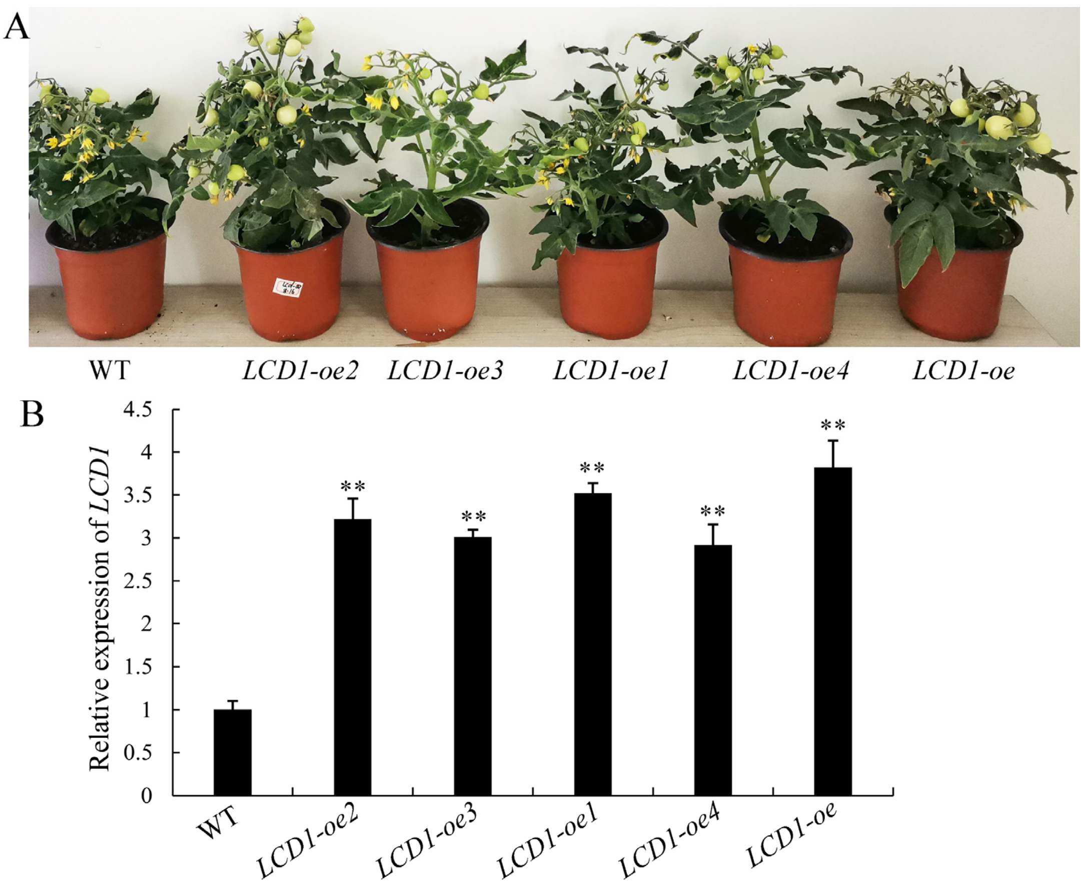

Figure S1. (A) Images of wild type tomato and five *LCD1* overexpression lines and (B) relative expressions of *LCD1* in wild-type and overexpression lines determined by RT-qPCR. Data are means of three biological replicates ± standard deviation (SD). The symbol ** above the columns stand for significant difference between wild type and overexpression lines (p<0.01).

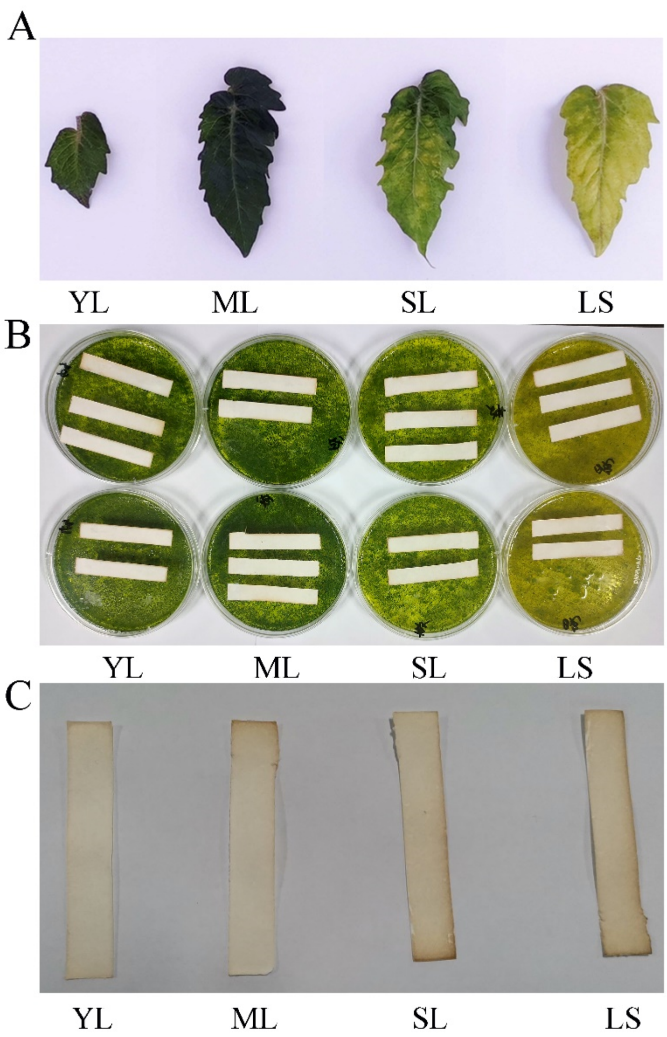

Figure S2. (A) Representative images of *Solanum lycopersicum* Micro-Tom leaves at different developmental stages, young leaves (YL), mature leaves (ML), senescent leaves (SL), and late senescent leave vs (LS). (B) The images of the detection of H_2S production in tomato leaves at different developmental stages by lead acetate H_2S detection strips (Sigma-Aldrich). The strips were attached to the inner part of the upper lid of the petri dishes. (C) The images of H_2S detection strips after two hours' detection in tomato leaves at different developmental stages.
